# Characterizing Non-Tuberculous Mycobacteria Infection in Bronchiectasis

**DOI:** 10.3390/ijms17111913

**Published:** 2016-11-16

**Authors:** Paola Faverio, Anna Stainer, Giulia Bonaiti, Stefano C. Zucchetti, Edoardo Simonetta, Giuseppe Lapadula, Almerico Marruchella, Andrea Gori, Francesco Blasi, Luigi Codecasa, Alberto Pesci, James D. Chalmers, Michael R. Loebinger, Stefano Aliberti

**Affiliations:** 1 Dipartimento Cardio-Toraco-Vascolare, University of Milan Bicocca, Respiratory Unit, San Gerardo Hospital, ASST di Monza, Via Pergolesi 33, 20900 Monza, Italy; paola.faverio@gmail.com (P.F.); annetta.stainer@gmail.com (A.S.); giu28686@hotmail.it (G.B.); s.zucchetti2@campus.unimib.it (S.C.Z.); edo.simonetta@gmail.com (E.S.); almx@libero.it (A.M.); alberto.pesci@unimib.it (A.P.); 2 Department of Internal Medicine, Division of Infectious Diseases, San Gerardo Hospital, ASST di Monza, Via Pergolesi 33, 20900 Monza, Italy; giuseppe.lapadula@gmail.com (G.L.); andrea.gori@unimib.it (A.G.); 3 Department of Pathophysiology and Transplantation, University of Milan, Cardio-Thoracic Unit and Cystic Fibrosis Adult Center, Fondazione IRCCS Cà Granda Ospedale Maggiore Policlinico, Via Francesco Sforza 35, 20122 Milan, Italy; francesco.blasi@unimi.it; 4 Villa Marelli Institute, Niguarda Ca’ Granda Hospital, 20122 Milan, Italy; luigiruffo.codecasa@ospedaleniguarda.it; 5 Scottish Centre for Respiratory Research, University of Dundee, Dundee DD1 9SY, UK; j.chalmers@dundee.ac.uk; 6 Host Defence Unit, Royal Brompton and Harefield NHS Foundation Trust, London, UK Imperial College London, London SW3 6NP, UK; M.Loebinger@rbht.nhs.uk

**Keywords:** non-cystic fibrosis bronchiectasis, non-tuberculous mycobacteria, pulmonary infection

## Abstract

Chronic airway infection is a key aspect of the pathogenesis of bronchiectasis. A growing interest has been raised on non-tuberculous mycobacteria (NTM) infection. We aimed at describing the clinical characteristics, diagnostic process, therapeutic options and outcomes of bronchiectasis patients with pulmonary NTM (pNTM) disease. This was a prospective, observational study enrolling 261 adult bronchiectasis patients during the stable state at the San Gerardo Hospital, Monza, Italy, from 2012 to 2015. Three groups were identified: pNTM disease; chronic *P. aeruginosa* infection; chronic infection due to bacteria other than *P. aeruginosa*. NTM were isolated in 32 (12%) patients, and among them, a diagnosis of pNTM disease was reached in 23 cases. When compared to chronic *P. aeruginosa* infection, patients with pNTM were more likely to have cylindrical bronchiectasis and a “tree-in-bud” pattern, a history of weight loss, a lower disease severity and a lower number of pulmonary exacerbations. Among pNTM patients who started treatment, 68% showed a radiological improvement, and 37% achieved culture conversion without recurrence, while 21% showed NTM isolation recurrence. NTM isolation seems to be a frequent event in bronchiectasis patients, and few parameters might help to suspect NTM infection. Treatment indications and monitoring still remain an important area for future research.

## 1. Introduction

Bronchiectasis represents a significant disease entity with increasing prevalence and substantial impact on patients’ morbidity and mortality, as well as healthcare utilization [1]. Chronic airway infection plays a key role in the pathogenesis of the disease sustaining a vicious cycle of inflammation and structural damage [2]. *P. aeruginosa* defines a specific clinical phenotype of bronchiectasis, and its presence is clearly associated with worse patient outcomes [3,4,5]. The most frequently-isolated bacteria in sputum from bronchiectasis patients include *H. influenzae*, *P. aeruginosa*, *M. catarrhalis* and *S. aureus*. Among other pathogens, recent reports demonstrated an increasing role of non-tuberculous mycobacteria (NTM) with a frequency ranging from one to 18% in bronchiectasis patients [6,7].

Anatomic alteration of the bronchi along with airway clearance impairment seem to be the primum movens of chronic NTM infection, although some authors have also speculated about a possible role of NTM in directly causing bronchiectasis [8,9]. Treatment in pulmonary NTM (pNTM) remains extremely challenging in bronchiectasis. These patients usually meet per se two out of three criteria for pNTM disease recommended by the 2007 American Thoracic Society (ATS)/Infectious Diseases Society of America (IDSA), regardless of the presence of NTM, having both respiratory signs/symptoms and radiographic abnormalities (bronchiectasis) [8]. Thus, translating current evidence and recommendations for the general population with pNTM disease to a specific population of bronchiectasis patients might not be fully appropriate. In view of this scenario, specific evidence on NTM infection in bronchiectasis is needed to help physicians in identifying patients with pNTM disease and treating them appropriately [10].

The objective of this study was to describe the clinical, functional and radiological characteristics of bronchiectasis patients with pNTM infection, as well as the diagnostic process, therapeutic options and outcomes. We also aimed at comparing the characteristics of bronchiectasis patients with pNTM with those with a chronic infection due to *P. aeruginosa* or other bacteria.

## 2. Results

### 2.1. NTM Infection and pNTM Disease in Bronchiectasis

**Figure 1:**
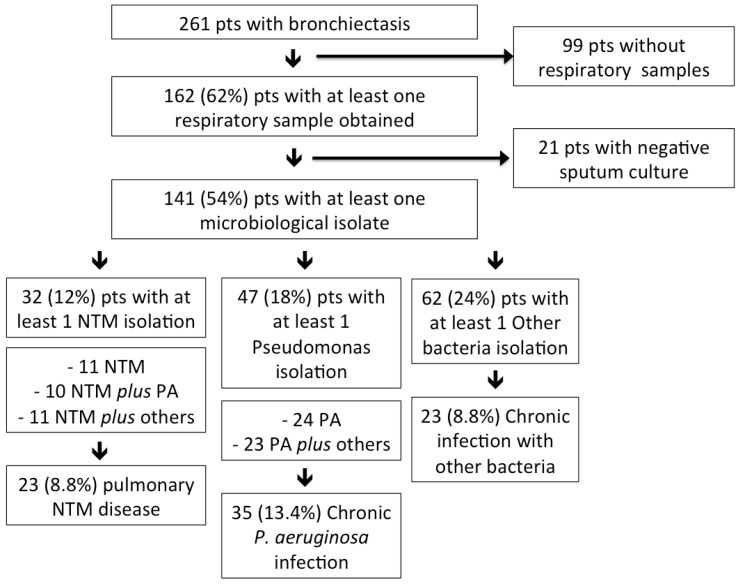
Division of the entire population according to microbiological isolations. PA = *P. aeruginosa*; NTM = non-tuberculous mycobacteria; pts = patients.

Among 261 bronchiectasis patients (median age: 69 years, 59% female) attending the clinic over the study period, 141 (median age: 69 years, 58% female) had a positive microbiology finding in the respiratory sample; see Figure 1. Among the entire study population, 136 (52%) patients underwent bronchoscopy; when considering only those with a positive respiratory sample, the percentage of patients who underwent bronchoscopy is as high as 61%. At least one NTM was isolated in 32 patients (23% among patients with an isolated pathogen and 12% among all bronchiectasis patients). The most common NTM were *Mycobacterium avium* complex (MAC) (24 patients, 17%), including 13 *M. avium* and 11 *M. intracellulare*, and *M. chelonae* (two patients, 1.4%); see Table 1. *M. gordonae* was isolated in four patients and was considered as a contaminant. Two NTM were isolated in one patient: *M. abscessus* spp. and subsequently *M. chelonae* (no treatment was initiated in this case). None of the NTM isolates were resistant to macrolides. NTM isolation was obtained from bronchoscopic specimens in 59% of the patients and from sputum samples in the remaining 41%. A co-infection with other bacteria was detected in 21 (66%) NTM patients, including *P. aeruginosa* (10 patients), *S. aureus* (eight methicillin-susceptible and one methicillin-resistant *S. aureus*), *H. influenzae* (six patients) and *M. catarrhalis* (two cases). A total of 22 (16%) patients had a nodular-bronchiectatic pattern for the high-resolution computed tomography (HRCT) scan, eight (5.7%) patients a cavitary pattern and two (1.4%) patients a bronchiectatic pattern. All patients, but four, had daily respiratory symptoms, either cough or sputum production.

Among the 32 patients with a NTM isolation, a diagnosis of pNTM disease according to the 2007 ATS/IDSA guidelines was reached in 23 (72%) cases: 18 subjects started antibiotic treatment; four patients refused any pharmacological treatment; while one patient had severe liver disease contraindicating antibiotic treatment. Among the nine patients in whom the ATS/IDSA diagnosis of pNTM disease was not reached, a lack of either the clinical (four cases) or microbiological criterion (five cases) was observed. All, but one, did not start a specific antibiotic treatment, but were monitored during follow-up. The only patient who started antibiotic treatment without reaching the ATS/IDSA criteria had a MAC isolation on sputum and a cavitary pattern on HRCT.

### 2.2. Characteristics of Bronchiectasis Patients with NTM

Among the entire study population, 23 (8.8%) patients belonged to the pNTM group, 35 (13.4%) to the *P. aeruginosa* group and 23 (8.8%) to the other bacteria group (including *H. influenzae* in 12 cases, *S. aureus* in four cases, *K. pneumoniae* in three cases, *M. catarrhalis* in two cases, *E. coli* and *S. pneumoniae* in one case). Four patients with both pNTM and *P. aeruginosa* were included in the pNTM group. Seven patients with concomitant chronic infection with other bacteria and either pNTM or *P. aeruginosa* were included in the pNTM or *P. aeruginosa* group, respectively; see Figure 1. Demographics, comorbidities, clinical, radiological, functional and laboratory data of the three study groups are reported in Table 2, and disease severity is summarized in Table 3.

Patients with pNTM were more likely to have cylindrical bronchiectasis and a “tree-in-bud” pattern, as well as a history of weight loss in comparison to patients with *P. aeruginosa*. Furthermore, patients with pNTM, including both those on active treatment and those in follow-up, showed a lower disease severity and a lower number of pulmonary exacerbations at one-year follow-up compared to patients with *P. aeruginosa*.

In order to limit the confounding effect of multiple pathogens isolated from the same patient, a subset analysis of patients with only NTM isolates (11 cases) vs. patients with NTM and other pathogens co-infection (12 cases) was performed. No differences between groups were identified in regards to all of the items evaluated in Table 2 and Table 3.

### 2.3. Treatment and Outcomes of Bronchiectasis Patients with pNTM

**Figure 2:**
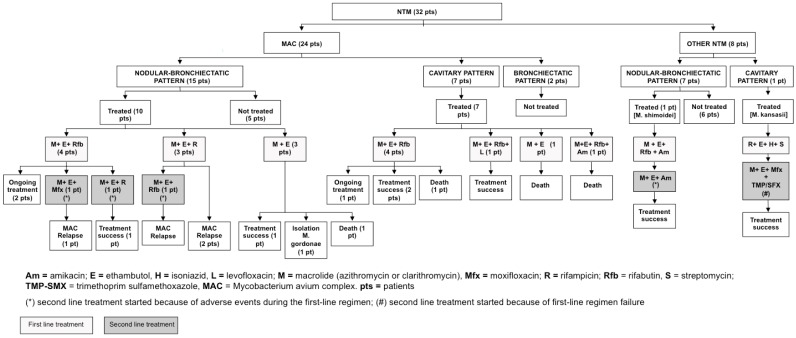
Therapeutic regimens and outcomes of adult bronchiectasis patients with pulmonary non-tuberculous mycobacteria disease according to the type of mycobacterium and the radiologic pattern.

Therapeutic regimens for pNTM are summarized in Figure 2. The regimen schedule was chosen according to both the isolated pathogen and the radiologic pattern after multidisciplinary discussion between pulmonologists and infectious disease physicians: six patients were on a three-times a week schedule and 13 patients were on a daily regimen (patients infected by NTM other than MAC and with a cavitary pattern on the HRCT scan were included in the latter group). All regimens included standard antibiotic doses adjusted for either renal or liver function in the case of insufficiency. All medications were given orally with the exception of amikacin and streptomycin. Median (IQR) treatment duration was 18 (14–18) months. Nine patients experienced adverse events due to the antibiotic treatment, including gastric intolerance (five cases), liver toxicity (two cases), thrombocytopenia (one case) and visual toxicity (one case). A second line regimen was initiated in five patients (26%) because of the presence of adverse events during the first line regimen in four cases and because of treatment failure in one case; see Figure 2.

Among pNTM patients who started treatment, 13 (68%) showed a radiological improvement. Among them, seven also experienced culture conversion after treatment with no recurrence (treatment success). Median (IQR) duration of treatment for pNTM before culture conversion was 3 (2–4.5) months. Three (16%) patients were still on active first line treatment at the time of the present analysis. A total of four (21%) MAC patients had MAC isolation recurrence after treatment. One MAC patient had a new isolation of *M. gordonae* on one sputum sample after treatment that was considered contaminant. Median (IQR) duration of treatment for pNTM before culture conversion was 9 (5–16) months. Four (21%) patients died during NTM therapy due to causes not directly related to pNTM: two were not related to respiratory diseases, and two were caused by lung cancer. No differences were found between groups in regards to solid cancer rates. Among the four patients who refused or postponed treatment, none died. No statistically-significant differences were detected among groups in regards to all-cause mortality at one-, two- and three-year follow-up; see Table 3.

Four out of the five pNTM patients who did not receive treatment showed no radiological progression within the study period. They had a median (IQR) one-year exacerbations and hospitalizations rate of 2 (0.5–3) and 0 (0–1.5), respectively. Three cases had also other pathogens isolated on respiratory samples: *P. aeruginosa* (two cases), *M. catarrhalis* (one case), *H. influenzae* (one case) and methicillin-resistant *S. aureus* (one case).

Patients in the *Pseudomonas* and other bacteria groups also received long-term antibiotic therapy: patients in the *Pseudomonas* group received long-term macrolide therapy in five cases (16%) and long-term inhaled antibiotic therapy in four cases (13%) during the study period. Patients in the other bacteria group received long-term macrolide therapy in two cases (13%) and long-term inhaled antibiotic therapy in one case (6%) during the study period.

## 3. Discussion

This study shows that NTM are isolated in 12% of adult patients with bronchiectasis, while a specific diagnosis of pNTM disease requiring treatment is reached in 8.8% of them. When considering patients with at least one isolated pathogen, the prevalence of NTM is as high as 23%. MAC is the most frequent mycobacteria, while a co-infection with other bacteria is present in the majority of the patients (66%), including *P. aeruginosa* in almost one third of them. Patients with pNTM are more likely to have cylindrical bronchiectasis and a “tree-in-bud” pattern on HRCT, a history of weight loss, a lower disease severity and a lower number of pulmonary exacerbations compared to patients with chronic infection with *P. aeruginosa*. Among pNTM patients treated according to 2007 ATS/IDSA guidelines, only 37% achieved treatment success without recurrence, while 21% showed NTM isolation recurrence, and 21% died during treatment.

Our prevalence of 12% of NTM isolation and 8.8% of pNTM disease is in line with a recent meta-analysis reporting a 9.3% overall prevalence of NTM isolation in bronchiectasis patients worldwide and with other data coming from European cohorts and showing NTM isolations in 2%–10% of the subjects [7,11,12,13,14]. Notably, NTM isolation in our cohort was mainly obtained from bronchoscopic specimens. The high number of bronchoscopies we conducted according to our standard operating procedures might have increased the rate of NTM isolations and, consequently, of pNTM disease diagnosis (72% of cases with a NTM isolation). Differently from our study, Máiz et al. evaluated the prevalence of NTM isolation and pNTM disease in a cohort of 218 adult bronchiectasis patients in Spain considering only sputum samples [11]. The authors found a lower prevalence of both NTM isolation and pNTM disease (8.3% and 2.3%, respectively) compared to our cohort. These previous data suggest that the higher the number of bronchoscopies performed, the higher the probability of NTM isolation and, consequently, of pNTM disease diagnosis. In addition, the presence of a tree-in-bud pattern on HRCT scan and the inability to produce an adequate sputum sample were considered an indication to perform bronchial aspirate (BAS)/bronchoalveolar lavage (BAL) according to our standard operating procedures. This may also explain the high prevalence of pNTM disease diagnosis in our cohort. Our finding of MAC being the most frequent NTM in bronchiectasis also confirms previous data published by both Máiz and Mirsaeidi, who identified MAC in 50% and 80% of all NTM isolates, respectively [11,15]. Furthermore, our data show that in NTM patients, the most common co-infection is with *P. aeruginosa* (31%), and this is in line with previous experiences showing a prevalence ranging from 27% to −52% [14,16]. Finally, we also identified *S. aureus* and *H. influenzae* as other pathogens co-infecting bronchiectasis patients with NTM, as previously described [14].

Very scarce evidence supports experts’ opinion in suggesting when to perform culture for mycobacteria in bronchiectasis patients [17]. According to our results, few clinical parameters might be helpful in discriminating between NTM vs. chronic *Pseudomonas* infection in bronchiectasis. Key findings that should be investigated and might lead physicians to consider a patient at higher risk for NTM infection include weight loss, a tree-in-bud pattern and cylindrical bronchiectasis at HRCT scan. Similarly to our results, Máiz and colleagues reported that a low body mass index was independently associated with NTM isolation [11]. Notably, Koh et al. identified the presence of bronchiolitis, lobular consolidations and cavities as radiological findings related to pNTM in 105 bronchiectasis patients [18].

Although two thirds of our patients had radiological improvement during treatment, only 37% achieved culture conversion without recurrence, while 21% showed NTM isolation recurrence. Similar results with a treatment success rate of 40%–60% and high rates of NTM isolation relapse or re-infection (up to 50% of patients who completed treatment) have also been found in larger MAC cohorts [19,20,21]. According to these results, it seems that, despite a successful antibiotic course, a large percentage of bronchiectasis patients develop NTM recurrence.

In this scenario, the vicious cycle connected with bronchiectasis leading to impaired airway clearance and chronic airways infection could be considered one of the main risk factors for pNTM disease recurrence, together with other host (e.g., immunodeficiency) and environmental risk factors. We might identify two possible repercussions on patients’ management: from a diagnostic point of view, a higher relevance should be given to bronchiectasis severity, extension and radiological worsening during follow-up in the prognostic definition of the disease. This, along with clinical and microbiological criteria, could guide physicians in the decision making process whether to treat or not patients with pNTM disease. From a therapeutic point of view, physicians should keep in mind that a successful patient’s management requires therapeutic strategies for both NTM infection and bronchiectasis. Airway clearance techniques, bronchodilators if indicated and exacerbations/infections prevention should be started as soon as possible and continued after a specific antibiotic course. A long-term macrolide regimen for frequent exacerbators is probably the only therapeutic strategy that comes into conflict with proven or suspected pNTM disease, since macrolide monotherapy is contraindicated in this latter case.

Other interesting observations can be pointed out in our cohort of bronchiectasis patients with pNTM disease. Given the prolonged antimicrobial course with a number of potential side effects (developed in 47% of our patients), a significant proportion of patients decides to postpone or refuse treatment even when suggested otherwise (17% in our cohort, none died); similar results with treatment discontinuation or refusal in 10%–30% of patients have also been described in other cohorts [20,21]. Furthermore, the morbidity and mortality related to patients’ multiple comorbid conditions can often complicate short- and long-term outcomes [22], as described in our cohort where four patients died during pNTM treatment and one patient did not start antimicrobial therapy because of the severity of the comorbid conditions.

Although we present data from one of the largest cohorts of pNTM infection in adult bronchiectasis patients described so far in Europe, some limitations of our study should be acknowledged. On the one hand, the monocentric design limited our possibility to draw conclusions concerning the comparison of NTM patients with those with other chronic infections and impacts the generalizability of these and other results. Among four patients with pNTM disease who experienced recurrence after treatment of the same NTM species, we were not able to differentiate between true relapse vs. re-infection. On the other hand, the prospective nature of our study performed in a referral centre for bronchiectasis gave us the opportunity to work on a homogeneous cohort of patients with high quality data and with a complete clinical and microbiological history. Furthermore, a few possible confounders should be listed. Firstly, an important proportion of pNTM patients is on a prolonged antibiotic regimen with multiple drugs, including macrolides, which may have affected the exacerbation rate. Secondly, since all patients with a tree-in-bud pattern on HRCT scan without sputum production underwent bronchoscopy, the presence of tree-in-bud itself may self-select for NTM. Thirdly, some patients in the pNTM group had also other respiratory isolates, including *P. aeruginosa* (six cases) and other pathogens (six cases). One of the main limitations of the present study is the impossibility to perform statistical analysis on patients with only NTM infection and no other pathogen isolated due to the small sample size (11 patients had only NTM infection); therefore, in the NTM pulmonary disease group, four patients had both NTM pulmonary disease and *P. aeruginosa* chronic infection.

Future studies should focus on determining whether patients with NTM isolation and bronchiectasis may benefit from different diagnostic criteria to define pNTM disease. Finally, given the frequently unsatisfactory outcomes after treatment, further research is needed to evaluate whether patients with pNTM disease and bronchiectasis may require specific therapeutic regimens, schemes and durations of treatment.

In conclusion, the isolation of NTM seems to be a frequent event in bronchiectasis patients, especially among those with cylindrical bronchiectasis and a “tree-in-bud” pattern on HRCT, a history of weight loss, a low disease severity and a low number of pulmonary exacerbations. Treatment indication in this specific population, as well as monitoring patients’ response still remain important areas for future research.

## 4. Materials and Methods

### 4.1. Study Design

This was a prospective, observational study of adult patients with bronchiectasis attending the outpatient clinic at the San Gerardo Hospital, Monza, Italy, from 2012–2015. Consecutive patients aged ≥18 years with a diagnosis of bronchiectasis on HRCT scan in a stable state were recruited. Patients with cystic fibrosis or traction bronchiectasis due to pulmonary fibrosis were excluded. The Institutional Review Board of the San Gerardo Hospital approved the study (ethical permission code: 234, 30 September 2013), and patients signed an informed consent.

### 4.2. Data Collection and Microbiological Analysis

At the time of clinical assessment, all patients underwent the same comprehensive diagnostic work-up as recommended by the 2010 British Thoracic Society (BTS) guidelines [17]. Demographics, comorbidities, disease severity, respiratory symptoms, microbiology, radiological, functional and laboratory findings in the stable state, long-term treatments and outcomes (including exacerbations, hospitalizations and mortality) during a three-year follow-up were recorded. HRCT imaging was performed using a 64-slice CT scanner. Sequential scanning was performed at maximal inspiration from the apex to the diaphragm using 1-mm contiguous slices (1-mm set), while patients were in the supine position. Images were reviewed by a consultant radiologist with 15-year experience of reporting HRCT and a consultant respiratory physician with a major interest in bronchiectasis in order to define nodular-bronchiectatic vs. cavitary vs. bronchiectatic patterns. They etiology of bronchiectasis was evaluated as previously described [23]. The severity of bronchiectasis was evaluated according to the bronchiectasis severity index (BSI) [24,25].

All bacteriology, including culture for both bacteria and mycobacteria, was performed on either spontaneous sputum (for patients with a productive cough) or BAS/BAL samples. BAS/BAL were collected in patients showing a HRCT appearance of a tree-in-bud pattern without productive cough. Murray–Washington criteria for sputum quality were used in all cases, with all samples having less than 10 squamous cells and more than 25 leukocytes per low-power microscope field.

### 4.3. Study Definitions and Outcomes

pNTM disease was defined according to the 2007 ATS/IDSA guidelines as the presence of both clinical (pulmonary symptoms and radiographic abnormalities) and microbiological criteria (NTM positive culture results from at least two separate sputum samples or one bronchoscopic specimen) [8]. Chronic infection was defined by the isolation of potentially-pathogenic bacteria in sputum culture on two or more occasions, at least 3 months apart over a 1-year period [26]. A bronchiectasis exacerbation was defined as a clinical diagnosis of exacerbation for which antibiotics were prescribed in the presence of at least one (and usually more than one) of the following symptoms: increasing cough, increasing sputum volume, worsening sputum purulence, worsening dyspnoea, increased fatigue/malaise, fever and haemoptysis [17].

Study outcomes included exacerbations, hospitalizations and all-cause mortality at one-year follow-up, as well as all-cause mortality at two- and three-year follow-up.

### 4.4. Study Groups

The study population was divided according to the presence of bacteria and NTM: (1) patients with pNTM disease; (2) those with chronic *P. aeruginosa* infection; and (3) those with chronic infection due to bacteria other than *P. aeruginosa*. Patients with both pNTM disease and chronic infection with either *P. aeruginosa* or other bacteria were included in the pNTM disease group, whereas patients with a chronic infection with both *P. aeruginosa* and other bacteria were included in the *P. aeruginosa* group.

### 4.5. Statistical Analysis

Data were analysed using SPSS 21.0 for MAC OS (SPSS Inc., Chicago, IL, USA). Characteristics of the population (including respiratory symptoms), radiological features, pulmonary function tests (PFTs) and microbiological isolation, as well as study outcomes were considered for statistical analysis. Continuous variables are expressed as median (interquartile range (IQR) 25th–75th percentile). The difference of median (IQR) was evaluated by the Wilcoxon–Mann–Whitney U two-sample test. Categorical data are expressed as frequencies and percentages and compared using the chi-square or Fisher exact test where appropriate. All tests were 2-tailed, and a *p*-value <0.05 was considered statistically significant.

## Figures and Tables

**Table 1:** Non-tuberculous mycobacteria isolated in the study population.

Non-Tuberculous Mycobacteria		
*Mycobacterium avium* complex		24
–	*M. avium*	13
–	*M. intracellulare*	11
*M. gordonae*	–	4
*M. chelonae*	–	2
*M. kansasii*	–	1
*M. abscessus* spp.	–	1
*M. shimoidei*	–	1

**Table 2:** Demographics, comorbidities, radiological characteristics, symptoms, pulmonary function and laboratory data according to the three study groups: pulmonary non-tuberculous mycobacteria disease (pNTM); chronic infection with *P. aeruginosa* (*Pseudomonas*) and chronic infection with bacteria other than *P. aeruginosa* (other bacteria).

Variables	pNTM (*n* = 23)	*Pseudomonas* (*n* = 31)	Other Bacteria (*n* = 16)	*p*-Value
Demographics	–	–	–	–
Male, *n* (%)	10 (44)	15 (48)	6 (38)	0.79
Age, median (IQR)	70 (62–76)	72 (66–76)	60 (51–71)	0.77
BMI, median (IQR)	20.6 (18.7–25.1)	22.6 (21.6–25.2)	21.4 (18.1–27.2)	0.15
BMI < 18.5, *n* (%)	3 (19)	4 (13)	4 (27)	0.68
Prior tuberculosis, *n* (%)	4 (22)	3 (10)	1 (6)	0.39
Either smoker or ex-smoker, *n* (%)	11 (65)	13 (42)	6 (38)	0.23
Comorbidities, *n* (%)	–	–	–	–
Chronic obstructive pulmonary disease	6 (35)	13 (42)	2 (13)	0.76
Asthma	1 (6)	3 (10)	4 (25)	1
Sinusitis	1 (6)	5 (16)	6 (38)	0.65
Cardiopathy	4 (25)	14 (45)	7 (44)	0.22
Arterial hypertension	6 (38)	17 (55)	6 (38)	0.36
Angina	0	2 (7)	1 (6)	0.54
Prior stroke	0	1 (3)	0	1
Vasculopathy	2 (13)	4 (13)	0	1
Atrial fibrillation	0	4 (13)	0	0.28
Valvulopathy	1 (6)	6 (19)	2 (13)	0.39
Congestive heart failure	0	2 (7)	0	0.54
Pulmonary hypertension	0	2 (7)	2 (13)	0.54
Diabetes	3 (18)	6 (19)	1 (6)	1
Liver disease	1 (6)	1 (3)	0	1
Cirrhosis	1 (6)	1 (3)	0	1
Chronic renal failure	0	2 (7)	0	0.54
Neurological disease	0	2 (7)	1 (6)	0.54
Rheumatological disease	0	5 (16)	1 (6)	0.15
Vasculitis	0	1 (3)	1 (6)	1
Gastroesophageal reflux disease	5 (31)	10 (32)	8 (50)	1
Immuno-deficit	0	1 (3)	4 (25)	1
Solid cancer	7 (39)	6 (19)	2 (13)	0.18
Haematological malignancy	0	2 (7)	1 (6)	0.54
Radiologic characteristics, *n* (%)	–	–	–	–
Cylindric	13 (87) *	16 (52) *	12 (75)	0.048
Cystic	2 (13)	11 (38)	3 (19)	0.16
Varicose	0	2 (7)	1 (6)	0.54
Tree-in-bud pattern	13 (57) *	7 (23) *	7 (44)	0.011
Symptoms, *n* (%)	–	–	–	–
Daily cough	9 (56)	22 (71)	8 (50)	0.35
Daily sputum	5 (31)	18 (58)	10 (63)	0.13
Haemoptysis	4 (25)	4 (13)	5 (31)	0.42
Dyspnoea	9 (56)	18 (58)	9 (56)	1
Recurrent pneumonias	1 (6)	0	5 (31)	0.34
Weight loss	6 (38) *	3 (10) *	3 (19)	0.045
Asthenia	7 (44)	16 (52)	7 (44)	0.76
PFTs	–	–	–	–
FEV_1_ %, median (IQR)	85 (56–100)	61.5 (49–81)	82 (52–100)	0.074
Laboratories collected during stable phase		–	–	–
WBC × 10^3^/mL, median (IQR)	6.4 (5.4–7.7)	7.4 (5.8–8.6)	7.13 (5.48–9.61)	0.19
CRP mg/mL, median (IQR)	0.57 (0.15–2.3)	0.5 (0.26–1.38)	0.97 (0.17–3.18)	0.76

* *p* < 0.05 (pNTM vs. *Pseudomonas*). BMI = body mass index, FEV_1_ = forced expiratory volume in 1 s, WBC = white blood cells, CRP = C-reactive protein, PFT = pulmonary function test.

**Table 3:** Disease severity and outcomes according to the three study groups: pulmonary non-tuberculous mycobacteria disease (pNTM); chronic infection with *P. aeruginosa* (*Pseudomonas*) and chronic infection with bacteria other than *P. aeruginosa* (other bacteria).

Bronchiectasis Severity and Outcome	pNTM (*n* = 23)	*Pseudomonas* (*n* = 31)	Other Bacteria (*n* = 16)	*p*-Value
BSI, median (IQR)	7 (6–10) *	12 (9–15) *	6 (4–9)	0.004
One-year exacerbations, median (IQR)	0 (0–2) *	1 (0.75–3.25) *	0 (0–2)	0.043
One-year hospitalization (at least one/y), *n* (%)	2 (9)	5 (16)	0	0.94
One-year mortality, *n* (%)	1 (4.3)	1 (3.2)	0	0.76
Two-year mortality, *n* (%)	2 (8.7)	2 (6.5)	0	1
Three-year mortality, *n* (%)	4 (17.4)	4 (12.9)	0	0.9

* *p* < 0.05 (pNTM vs. *Pseudomonas*). BSI = bronchiectasis severity index.

## Abbreviations

NTM	non-tuberculous mycobacteria
pNTM	pulmonary NTM
ATS	American Thoracic Society
IDSA	Infectious Diseases Society of America
MAC	*Mycobacterium avium* complex
HRCT	high-resolution computed tomography
BAS	bronchial aspirate
BAL	bronchoalveolar lavage
BTS	British Thoracic Society
BSI	bronchiectasis severity index
